# Spatial Mobility Change Among Older Chinese Immigrants During the COVID-19 Pandemic: The Role of Physical, Social, and Virtual Environmental Factors

**DOI:** 10.3390/ijerph22030406

**Published:** 2025-03-10

**Authors:** Minhui Yang, Lu Wang, Lixia Yang, Jie Yu, Dongmei Chen, Miao Wang, Haoran Dong, Jingheng Yan

**Affiliations:** 1 Dalla Lana School of Public Health, University of Toronto, Toronto, ON M5S 1A1, Canada; mh.yang@mail.utoronto.ca (M.Y.); haoran.dong@torontomu.ca (H.D.); 2 Department of Geography and Environmental Studies, Toronto Metropolitan University, Toronto, ON M5B 2K3, Canada; jie.yu@mcgill.ca; 3 Department of Psychology, Toronto Metropolitan University, Toronto, ON M5B 2K3, Canada; lixiay@torontomu.ca (L.Y.); elisawang26@gmail.com (M.W.); 4 Department of Geography and Planning, Queen’s University, Kingston, ON K7L 3N6, Canada; chendm@queensu.ca (D.C.); 17jy55@queensu.ca (J.Y.)

**Keywords:** spatial mobility, older immigrants, virtual environment, neighbourhood and health, Chinese, COVID-19 pandemic, Chinese immigrants

## Abstract

Background: Vast spatial mobility changes happened globally during the COVID-19 pandemic, profoundly affecting older adults’ well-being and active aging experience. This study aims to examine how the virtual environment and cyberspace, in conjunction with the physical and social neighbourhood environments, influence outdoor activities and spatial mobility for older immigrants. Methods: Four online focus groups were conducted with 25 older Chinese immigrants aged 65 and over in the Greater Toronto Area, Canada. The focus groups explored coping strategies during the pandemic and spatial mobility patterns related to different activity types such as grocery shopping, leisure activities and physical exercises, social and familial activities, and healthcare. Qualitative thematic analysis was conducted guided by the neighbourhood and health theoretical framework. Results: The overall engagement of older Chinese immigrants in various types of outdoor activities declined drastically and the spatial mobility pattern was complex. This change was shaped largely by the intersecting physical/built (e.g., residential conditions, access to public spaces), social (e.g., social support, interpersonal cohesion) and virtual (e.g., online communities and internet-based resources) environmental factors, as well as individual risk perceptions towards COVID-19 and public health interventions during the pandemic. Conclusions: Virtual environment emerged as an important domain that compensates for the heavily reduced spatial mobility of the group during the pandemic. It functioned as a vital channel for older Chinese immigrants to sustain the necessary leisure, social, and healthcare-related activities and maintain well-being during the pandemic. The study provides implications for addressing neighbourhood-level factors in policymaking and implementing initiatives to enhance active ageing experience of older Chinese immigrants.

## 1. Introduction

Neighbourhood environment factors, such as those related to the physical and social features in residential areas, play essential roles in shaping the spatial mobility patterns and health behaviour of older Chinese immigrants globally, especially among those whose lives are more constrained to the immediate surroundings of their residences [1,2,3]. The everyday living of older Chinese immigrants in Canada was vastly affected by the COVID-19 pandemic. During the pandemic, the virtual environment emerged as a vital domain where older adults fulfill everyday living and well-being-related needs [4,5]. This new development has its unique influences on the spatial mobility and well-being of older Chinese immigrants across the globe, including Canada. Building upon the theoretical framework of neighbourhood and health, this study adopts a qualitative approach to explore the impacts of physical, social, and virtual environment factors on older Chinese immigrants’ spatial mobility changes during the pandemic in the Greater Toronto Area (GTA), the most culturally diverse and largest urban centre of Canada.

### 1.1. Background

The COVID-19 pandemic massively altered spatial mobility patterns of individuals, especially in urban settings. Mobility, in general, refers to the “physical movements of individuals” [6]. In this study, spatial mobility is defined as the ability and extent of physical/spatial movements that individuals make outside of their residential space for purposes such as social, shopping, health and physical activities, and other daily functions.

During the pandemic, apart from government-enforced restrictions, spatial mobility patterns of general populations were strongly altered by neighbourhood- and individual-level factors, such as residential stability [7], cultural backgrounds [8], social and economic status [9], and immigration status [10]. Long and Ren [7] discovered that some neighbourhood-level factors had direct effects on residents’ spatial mobility. Neighbourhoods with better residential stability show stronger community connections, and their residents are more likely to follow local lockdown policies to reduce spatial mobilities. Communities with higher cultural diversity show less mobility reduction, implying that people of different cultural backgrounds might hold distinct risk perceptions and react to governmental lockdown measures differently [7].

Spatial mobility is critical in shaping the well-being, quality of life and active ageing experience of older adults [11,12]. The voluntary and involuntary spatial mobility reductions had vast impacts on the health and well-being of older adults at the physical, mental, and social levels. Spatial mobility can be deemed a social determinant of health, as previous theoretical and empirical studies have revealed that spatial mobility reductions have direct negative impacts on the health outcomes of older adults [13,14]. Such effects were proven during the pandemic when spatial mobility reduction happened abruptly. Studies by Choe et al. [15] and Dobbie et al. [16] reveal significant mobility reduction among older adults, leading to a large decrease in physical activity. Another two studies from Singapore and Canada showed that the social connections and activities of older adults were further limited during the pandemic due to reduced mobility, which in turn impacted the older adults’ mental and social well-being [17,18].

The reduction in spatial mobility during the pandemic urged people, including older adults, to explore virtual channels as a substitute to meet their everyday living and well-being needs. The virtual strategies include but are not limited to online group activities, virtual medical consultations, social media, messaging, and online video calls [5,17,19,20,21]. Maintaining social connections through phone calls, messaging, or social media and accessing information and resources online have, to some degree, empowered older adults to feel a sense of connection as well as autonomy during lockdown periods [17,22]. Additionally, online delivery services and community-organized volunteer delivery were vital assistance to many older adults, providing them with access to essential supplies [5]. However, from the onset to the end of the pandemic, it is reported that older adults have continued to encounter issues while using virtual spaces, including accessibility challenges related to hardware and internet access, as well as cognitive barriers in understanding new digital technologies [23,24].

Ontario, Canada, was hit by the COVID-19 pandemic at the beginning of 2020, followed by fast-deployed public health measures including but not limited to limiting the size of gatherings and access to public indoor places [25]. Despite these lockdown measures, older adults disproportionately suffered from COVID-19 infection and a high mortality rate [26]. The preventive measures and information on the high mortality rates could lead to fundamental changes in spatial movement patterns of older adults, including older Chinese immigrants, leading to more challenges regarding everyday living. Previous studies suggested strong negative impacts of language and other sociocultural barriers on the mobility, social integration, and quality of life of older immigrants [27,28]. During the pandemic, older Chinese immigrants also experienced rapidly rising anti-Chinese discrimination [29], social isolation [30], and barriers to accessing healthcare services [31].

Previous studies explored the roles of sociocultural factors in changing older Chinese immigrants’ spatial mobility. However, the impacts of the residential neighbourhood environment remain a greatly understudied area. Moreover, while the virtual environment has become an increasingly critical domain for human interactions and activities, it remains unclear, in the Canadian context, how virtual space influences the spatial mobility, daily routine, and well-being of older immigrants. In these contexts, the current study aims to address the following research questions:

1. What were the key spatial mobility patterns (characteristics, trip purposes and changes) among older Chinese immigrants during the pandemic?
2. How did the virtual environment, in conjunction with physical and social environmental factors and individual characteristics, influence their spatial mobility patterns?

### 1.2. Conceptual Framework

Building on the neighbourhood and health theoretical framework, this study develops a conceptual framework to examine how spatial mobility is shaped by related physical, social and virtual environments. Neighbourhood environment is seen as an independent factor that has enduring features and directly affects people’s health behaviours and outcomes regardless of their personal living circumstances and choices [32,33]. The neighbourhood environment is categorized into physical and social aspects, which have influences on and can be translated into each other [32,34]. The physical environment includes geographical areas containing natural and built features [3,27]. Physical factors such as land use patterns and the ecological structure of routine activities are proven to have significant impacts on health behaviours, as they directly affect people’s accessibility to facilities for health-related purposes [33]. Regarding older Chinese immigrants, it is revealed that health and well-being outcomes are directly related to physical environmental factors such as transportation, accessibility to places for entertainment, and physical activities [3]. The social dimension of the environment includes a range of factors such as social cohesion, interpersonal connections (friendship, familial support), norms, and racial composition [33,35]. For Chinese immigrants, especially the ones who lack local language proficiency, social networks mainly consist of familial relations and community in their residential neighbourhood, which both affect their health and well-being outcomes [3].

In recent studies, the virtual environment has emerged as a new essential component constructing the living contexts of people [36,37] and may further expand the definition of the neighbourhood environment. The virtual environment/cyberspace allows for obtaining information as well as making connections, achieving collaborations, and building communities [36]. The conceptual framework considers all three dimensions of environment (physical, social, and virtual) as important intersecting factors that play different roles in affecting older Chinese immigrants’ ability in arranging outdoor activities such as grocery shopping, leisure activities and physical exercises, social and familial activities, and healthcare.

## 2. Data Collection and Analysis Methods

This paper draws from a larger research project that examines the impact of the COVID-19 pandemic on the Chinese community in the Toronto area of Canada. The study received a full ethics approval from the Research Ethics Boards of the authors’ institutions. Participants in this study were included if they met the following criteria: self-identify as Chinese and were born outside of Canada; have lived in Canada for more than six months; can understand and consent to participation; live in the Greater Toronto Area (GTA); and are over the age of 65. The participants of the larger research project were grouped by age and allocated into ten focus groups. Four focus group (FG) interviews were conducted with 25 Chinese immigrant older adults, recruited through multiple channels, including community-based organizations and community health centres that provided services for local older adult populations; messages on social media platforms and in chat groups frequently used by older Chinese immigrants; posts in local newspapers with older adult readers; and flyers disseminated in senior housing apartment buildings. These recruitment strategies enabled us to approach older Chinese immigrants from diverse backgrounds, residential locations, and living conditions.

Structured FG interviews were conducted between December 2020 and February 2021. Each FG interview took between 1 and 1.5 h. The FG interviews followed pre-designed questions, centred around exploring mobility patterns during the pandemic and related aspects of well-being, prevention measures, neighbourhood contexts, and coping strategies for spatial mobility reduction. All FGs were conducted in Mandarin, the groups’ preferred and first language. Two female bilingual (Mandarin and English) research team members conducted the interviews together to facilitate discussions and observe the group dynamics. The similar cultural and linguistic background shared between the interviewers and the focus group participants helped with developing a rapport with the participants, who were part of a vulnerable group in society. Such rapports had a positive effect, producing a rich, detailed conversation in focus group discussions based on empathy and understanding of sociocultural-based nuances [38,39]. Due to public health interventions in place on social distancing, all FGs were conducted online via Zoom. The nature of online FG rendered fewer interactions among participants during the interviews. Nevertheless, the online FGs managed to include participants from more diverse residential locations since no travel was required for participation. Each FG session contained participants who resided in different areas of the GTA. Data saturation was achieved (reoccurring ideas and similar incidents and experiences were identified) after finishing the four FG interviews. The interviews were recorded and transcribed verbatim in Chinese by bilingual research team members.

The data analysis was carried out by the bilingual team members, who first used Mandarin Chinese to capture the nuances of participants’ accounts before translating important excerpts of transcripts into English for in-depth analyses. More specifically, a deductive thematic analysis method [40] was applied to the data analysis. Derived from the study’s conceptual framework, a codebook was developed prior to the coding process, including the following themes or parent codes: “built/physical environment”, “social environment”, “virtual environment”, “mobility patterns and changes”, and “individual factors”. The detailed codebook structure and definitions of codes were further revised iteratively during the coding process. The coding process was conducted by three team members in NVivo separately, with the same codebook and weekly meetings to triangulate the coded transcripts and communicate coding strategies. Meetings with other team members were also held weekly to discuss the unsolved discrepancies in the coding. During each meeting, the team members collectively revised the structures and definitions of codes and coding strategies to resolve discrepancies and achieve intra-team consistency. All the team members were English–Mandarin bilingual Chinese living in Canada. The sociocultural-based nuanced and implicit meanings of the participants’ narratives were also addressed during the meetings.

Table 1 summarizes the socio-demographic characteristics of the FG participants. A majority of the participants lived with their spouse during the pandemic, while a certain amount (24%) of the participants lived alone and reflected their unique concerns and experiences of spatial mobility. A majority of the participants (76%) had annual income less than 30,000 Canadian dollars, significantly less than the average income of the GTA residents at 45,200 per year [41]. The participants represented the living conditions and spatial mobility of a variety of residential locations across the GTA, including downtown, inner suburban (e.g., North York and Scarborough), and outer suburban areas (e.g., Thornhill and Mississauga). Most of the participants (80%) had received education above the secondary level.

## 3. Results

The study revealed that during the pandemic, the older Chinese immigrant participants experienced noticeable changes in spatial mobility pertaining to their everyday living- and well-being-related activities. The changes were directly and synergistically affected by intertwining factors of physical, social, and virtual environments, as well as their individual circumstances. Particularly, the functions, strengths, and limitations of the newly emerged virtual environment were extensively discussed by participants, as many gradually shifted to online activities during the pandemic. The findings are summarized below.

### 3.1. Key Changes in Spatial Mobility Patterns During Pandemic

The older Chinese immigrants in this study were engaged in outdoor spatial activities primarily for purposes related to everyday living, such as grocery shopping, leisure and physical activities, social and familial activities, and healthcare. During the pandemic, most of the participants heavily reduced all types of outdoor activities due to two main overarching individual-level factors: their relatively high risk perceptions towards COVID-19 and their willingness to comply with governmental public health guides. First, the participants overwhelmingly considered outdoor activity reduction and self-quarantine as the most effective measures to eliminate the risks of contracting COVID. Some described their drastic reduction in outdoor activities to zero, that is, “not going out at all”. These older immigrants considered themselves as an extremely high-risk group due to their older age, existing chronic health conditions, potential severe COVID-related health consequences, and caretaking burdens on their family members once infected with COVID-19. As one participant reflected,

“*The children worried that if we got infected… It is troublesome for the children, right? So we decided to stay at home. It is the best for the children. It is helpful (for them)*”

(FG 2, P1)

Second, the participants showed a strong tendency to comply with the prevention measures introduced by the governments. Since the public health recommendations mentioned reducing social activities and gatherings, these older immigrants tended to perceive going outside and sustaining outdoor activities as infringing governmental orders and causing chaos in society. In this circumstance, they wanted to “*collaborate with the government and the state to carry out some very specific work*” *as* “*Lao Bai Xing (老百姓, translated as ordinary people), are members (of the larger society)*” (*FG 3*, *P1*).

However, not all participants consistently conducted self-quarantine during the pandemic; some managed to maintain a certain level of outdoor activity despite their worries about contracting the COVID-19 virus. Nevertheless, their spatial behaviours and extent of spatial mobility were extensively altered to reduce the risks of transmission. As revealed in the FGs, the main purposes for their outdoor mobility included grocery shopping, leisure activities and physical exercises, social and familial activities, and doctor visits. For each trip purpose, spatial mobility patterns had changed in distinctive ways compared to before the pandemic. Table 2 summarizes the major changes in spatial mobility patterns for different trip purposes, compared with the participants’ pre-pandemic spatial routines.

Some leisure activities, physical exercises, social activities, and healthcare services did not necessarily require material exchange or embodied experiences, so older Chinese immigrants extensively chose to use virtual channels as a substitute as the pandemic continued. In this way, they could keep being aligned with their high-risk perceptions and tendencies of staying indoors. In this circumstance, the reduced spatial mobility often did not always lead to reduced participation in well-being-related activities or worsened quality of life, as some older immigrants managed to sustain the activities through online channels. However, whether they could successfully shift the activities online depended on the provision and accessibility of resources and communities in the virtual environment. The following subsections highlighted how various factors relating to the physical, social, and virtual dimensions of the environment have affected older Chinese immigrants’ spatial mobility patterns.

### 3.2. Physical Environmental Factors Influencing Spatial Mobility

More specifically, physical environmental factors, including residential conditions and proximity to outdoor public space such as greenspace and retail strip malls were found to affect the decision-making of spatial mobility among the participants. Regarding the influences of residential conditions, there existed a clear distinction in mobility patterns between those who lived in detached houses and those in high-density apartment buildings. Both single-house and apartment-building residents expressed a need for going outdoors during the pandemic. Residents of detached houses were able to use their front and backyards for basic needs of physical exercise by simply getting out of the enclosed indoor space without facing the risks of close contact with others. For apartment building residents, the situation was more complicated. As their indoor living spaces were limited, apartment building residents reported a strong desire to go outdoors for leisure and well-being-related purposes. On the other hand, some residents had very high-risk perceptions towards COVID-19, and, therefore, decided to remain indoors and lost touch with the outdoor environment for considerable durations during the pandemic.

The primary reason for the high level of perceived risk among apartment dwellers was mainly related to using elevators and hallways upon leaving their units. Elevators and hallways were deemed enclosed spaces that engendered chances of close contact with others and risks of COVID transmission. One participant shared her concerns about going outside caused by the use of elevators, “*We needed to take elevators in this building, and I found the air very stale... Every time I went downstairs [in elevator] it felt like risking my life*” (*FG 4*, *P2*). It echoes another participant’s concerns: “*The elevators in this apartment building were kind of dangerous. Because you had no idea who lived in this building and whether they had COVID or not. So I normally don’t go outside*” (*FG 3*, *P6*). In this way, despite their more pressing needs for outdoor activities due to compact indoor space, many older immigrants residing in apartment buildings demonstrated less spatial mobility compared with those living in other low-density residential settings such as detached houses.

Proximity to outdoor public space, such as parks, plazas, community centres and public libraries, also affected the spatial mobility of older Chinese immigrant participants. The majority of the older participants did not own a car, nor could they still drive. Before the pandemic, many relied on family members to drive them to different places. Such outdoor activities were suspended during the pandemic because of worries about intra-familial transmission of the virus. Some participants stopped taking public transportation altogether due to perceived transmission risks on buses, streetcars, and subways. As one participant shared, “*Now we consider taking buses or cars (as risky behaviour), because they are enclosed spaces. So now I don’t dare to take buses*” (*FG1*, *P2*).

On the other hand, local indoor public spaces such as gyms, shopping centres, and community centres were mostly closed due to public health measures during the pandemic. Therefore, outdoor public spaces in the neighbourhood became essential facilities for leisure activities and physical exercises for many participants. Whether they would go outside largely depended on their accessibility to green space and other outdoor open areas, such as plazas near their residential locations. As one participant shared, “*we basically stayed at home all the time since the beginning of the pandemic. But when the weather was good, we would go to the park on the north (to our home) for a walk to strengthen our bodies*” (*FG4*, *P5*).

### 3.3. Social Environmental Factors Influencing Spatial Mobility

The identified social environmental factors influencing participants’ spatial mobility include intra-family care, family support, influences of social networks, behavioural cohesion in the neighbourhood, and racial discrimination. Intra-family care refers to the mutual attentiveness between older immigrants and their family members, especially their adult children, regarding the risk and prevention of COVID-19 infection. For some participants, their high risk perception and corresponding mobility reduction were not only for their personal well-being but also associated with concerns for their family members. Getting infected with COVID-19 meant for them to exert caretaking burdens on the family members. “*We need to not only worry about our own issues, but also our son’s. Because our son’s family and our family are together… Because it is impossible that our son doesn’t take care of us. So we are afraid of [getting infected]*” (*FG3*, *P4*).

Some participants were asked by their children to stay indoors to avoid transmission and keep safe, and their self-quarantine decision was made collectively with their adult children. One participant shared that “*our son calls us every day and tells us, don’t go out! Don’t go out! … We are almost 70, and our son told us that we were a high-risk population, so we need to pay special attention*” (*FG4*, *P2*).

Family support mainly refers to the support provided primarily by the participants’ adult children to sustain their everyday lives amid spatial mobility reduction. Self-quarantine and lockdown were made possible by relying on family support to fulfill basic needs, particularly grocery shopping and delivery. One participant shared that “*we never go outside for grocery shopping. During the pandemic, all the groceries were bought and delivered by my son and daughter-in-law on their way home from workplaces*” (*FG1*, *P4*).

Social networks also shaped older Chinese immigrants’ spatial mobility patterns during the pandemic. It was reflected in the FGs that reducing conventional in-person social activities since the onset of the pandemic was considered a necessary and obligatory communal action or decision, despite their feelings of loss and isolation when staying home. Most participants observed a high behavioural cohesion among their social networks regarding the decisions to cancel social events and in-person social activities such as gathering with friends and families and community-based group activities, without experiencing much resistance from their networks. As the pandemic perpetuated, in-person social activities were gradually replaced by online ones. One participant shared how her social group changed strategies for their weekly activities:

“*Our organization, (organization name) used to have group activities every week. We would get together for the activities. Now they are suspended and we have shifted online*”

(FG1, P4)

Neighbourhood behavioural cohesion was another social environmental factor that affected the older Chinese immigrants’ spatial mobility. Many older Chinese immigrants lived in ethnically and racially diverse neighbourhoods and observed different behaviours of mask-wearing and social distancing. Despite their high-risk perception and extensive use of personal protective equipment, some older Chinese immigrants felt unsafe walking outside when other residents were less cautious about prevention. Their spatial mobility correspondingly reduced in both frequencies and durations, and they deliberately avoided close contact with strangers in public spaces. Participants observed distinctive behavioural patterns of Asians and people of other races and ethnicities: “*These Westerners found freedom the most important, and they didn’t wear masks… When I passed by someone face-to-face on the street and they wore masks, I found it okay. Now when I am on the street seeing someone coming in my direction (without wearing a mask), I would go to the other side of the street*” (*FG 2*, *P2*).

Furthermore, racial discrimination, demonstrated through hostile attitudes toward mask-wearing Chinese individuals, contributed to complicating the situation at the beginning of the pandemic. On the one hand, many older Chinese immigrants felt it was imperative to wear a mask to protect themselves from contracting COVID-19; on the other hand, they worried that wearing masks would engender other people’s hostility. This quandary defeated the older Chinese immigrants’ willingness to go outside: “*when we went outside and met some neighbours, their faces were… Because at that time, people were saying Wuhan virus, China virus… people might be thinking if our Chinese all carried the virus*” (*FG3*, *P3*).

### 3.4. Emerging Use of Virtual Environment—As a Substitute and New Possibilities

The FGs revealed that the virtual environment, facilitated by websites and social applications (Apps), played an important role in shaping the overall experiences of older Chinese immigrants, including their spatial mobility during the pandemic. Prior to the pandemic, many participants had already used online channels to obtain information and socialize. During the pandemic, these virtual spaces became crucial for maintaining social connections, engaging in leisure and physical activities, and accessing healthcare services. With in-person interactions limited by social distancing and travel restrictions, communication Apps became vital and rapidly expanded in usage among older Chinese immigrants for accessing leisure and well-being-related resources and healthcare services. Table 3 highlights how the virtual environment influenced the daily lives of older Chinese immigrants during the pandemic in conducting different types of activities.

As summarized in Table 3, the virtual environment served as a substitute for in-person channels when different types of in-person outdoor activities were suspended. As discussed earlier, older Chinese immigrants reduced or cancelled outdoor activities and turned to online platforms due to factors related to risk perceptions, government guidelines, and the lack of in-person services. This shift was most notable in the category of leisure and physical activities, which normally provided structure, fulfillment, and social connections for older adults. It was discussed extensively during the FGs that online lessons and activities emerged during the pandemic, and they were very helpful in maintaining personal well-being. For example, one participant shared the meaning of participating in online group activities: “*I didn’t know what to do at home and found it quite painful… the time was too much… Later, our community started to hold many online activities, and then I slowly adapted to the current situation*” (*FG4*, *P2*).

Some other outdoor and in-person activities, because of their requirements for embodied experiences, could not be successfully adopted in online formats. As shown in Table 3, many older Chinese immigrants did not engage in online grocery shopping, as not being able to pick produce by hand was considered to lead to poor grocery and food quality. These irreplaceable aspects of in-person activities urged the Chinese immigrants to go outside, despite their high risk perceptions. The conflicting needs of acquiring living necessities and disease prevention sequentially generated distress and mental burdens on some older Chinese immigrants, especially the ones who lived in high-rise buildings and perceived high chances of transmission in the public spaces of their buildings. As one participant reflected, “*I hesitate every time… I feel like I am putting my life at risk when I go downstairs (by elevators)... I need to go outside, but I am so afraid. There are a lot of conflicts in my mind*” (*FG4*, *P2*).

Also, the absence of embodied interactions made the social, leisure, and physical exercise activities different since the vibes of physically being together were missing. As one participant reflected, “*Sometimes we see other people’s performance [on screen], and it is recorded. It looks like a cartoon (not real)*” (*FG2*, *P3*). The embodied experience was particularly imperative for healthcare services. Some older immigrants wanted to receive checkups and treatments on their bodies, which could not be achieved through virtual channels. For example, one participant shared, “*My leg has been in pain recently, and I want to have it checked by a doctor. But it was really hard to get an [in person] appointment*” (*FG4*, *P5*). In fact, many participants were reluctant to shift to virtual healthcare services, however, they had to, because of their perceived risk of transmission in clinical settings and the cancellation of in-person appointments.

Older Chinese immigrants’ access to the virtual environment was largely shaped by pre-existing social connections and communities, such as local centers, churches, and hobby groups. These social networks determined access to online social, leisure, and exercise activities. With in-person gatherings suspended due to public health measures, organizers shifted activities online via platforms such as Zoom and Google Meet. Those already involved in these groups before the pandemic adapted more easily to virtual spaces, reducing their need for outdoor activities and spatial mobility. As one participant reflected, “*Our life had been very simple, and the major daily schedule was participating in community activities. People got together, chatting and laughing. Now we have changed to online video meetings. It is like the format changed, but the contents did not*” (*FG 2*, *P2*).

The virtual environment provided new possibilities that could not be realized through in-person channels because of its translocal attribute. Some participants were actively engaged in many online activities across geographical limits. Virtual delivery of programs made it possible for people living in a different city, region, or country to transcend physical geographical barriers and participate in online classes and activities organized in another locale. As one participant shared, “*I was a XX engineer, and I was a member of the American XX engineering association. This year, I participated in its activities all online*” (*FG2*, *P4*). Although the engagement in virtual activities resulted in no spatial movement or mobility for the participants, it is important to note their ability to participate in activities and access resources tied to a distant geographic location, which was not an option to them pre-pandemic.

## 4. Discussion

Applying qualitative methods, this study reveals the older Chinese immigrants’ nuanced experiences in their engagement in outdoor activities and complex patterns of spatial mobility during the pandemic. Guided by the conceptual framework, the neighborhood and health theories provide the general analytical directions to explore the physical and social dimension of the environment [32,34], and the emerging virtual environment was identified as an additional dimension shaping older Chinese immigrants’ overall experiences amid mobility restrictions and reductions during the pandemic.

High risk perception toward COVID-19 was discussed by FG participants as the primary reason for the drastic spatial mobility reduction during the pandemic. For many older Chinese immigrants in Western countries, personal health and well-being are closely tied to their children’s lives, as Chinese families often have strong intergenerational relationships [42,43]. Many immigrated to Canada for family reunification, typically lacking English proficiency and knowledge of the healthcare system [44]. As this study shows, they often have no local support networks beyond family and rely heavily on their children for care during health challenges. Contracting COVID-19 not only meant illness but also the potential disruption of family routines and caregiving responsibilities. In the worst cases, if children cannot provide care, they may have to navigate the healthcare system alone, facing significant difficulties related to language, transportation and material resources. These challenges led many older immigrants to adopt extreme mobility restrictions, reflecting their vulnerable social positions and limited access to support outside the family.

However, carrying out the drastic reduction in outdoor mobility and constantly staying indoors rendered the older Chinese immigrants fewer chances and options for physical activities. To cope with these physical, mental, and social well-being related challenges, the older Chinese immigrants in the study developed alternative patterns of outdoor spatial mobilities. The patterns are mainly demonstrated as a significant reduction in frequency and duration of outdoor activities, rigorous use of personal protection equipment when going outdoors, practicing strict disinfection measures, shortening stay in indoor public spaces, and avoiding close contact with people outside home.

This study shows that apartment building and house residents had quite distinctive spatial mobility patterns. The apartment building residents considered them immediately entering an “area of risk” at the moment they stepped out of their units. The buildings’ elevators and hallways were considered enclosed indoor spaces with a high chance of meeting people who were less cautious about preventive measures and being exposed to the virus. This unique challenge faced by apartment building residents is worth further investigation in preparation for future pandemic outbreaks. It has been observed that during the pandemic, high density of residents, clustered living conditions, and lack of designated preventive measures impacted the health and well-being of senior housing residents [45,46]. In the case of older adults, including older immigrants, structural factors also come into play to affect spatial mobility and well-being. In Canadian settings, senior housing is typically government-subsidized and provided to low-income older adults, many of whom are financially vulnerable and have a higher prevalence of multiple health conditions than the general older population [45]. Our study further demonstrates that the clustered living conditions usually led to the older Chinese immigrants’ concerns about close contact with others in the “area of risk”. This concern was a subjectively felt barrier to their spatial mobility, outdoor activities, and overall well-being.

The older Chinese immigrants’ use of the virtual environment shows some unique characteristics in this study. First, the older Chinese immigrants who more actively shifted to online channels often had already been deeply involved in social and hobby groups prior to the pandemic. On the other hand, older immigrants with fewer social connections might take longer and face more barriers in learning about the technologies and finding suitable online groups and activities aligned with their interests and tastes. Future research can take a deeper look into the social aspects of older immigrants’ online behaviours, how online activities reshape their social connections and networks, and how the virtual and social environmental factors work together to affect older immigrants’ spatial mobility and well-being. The second unique feature is related to the boundaryless nature of online spaces. Virtual environment allowed participants to access information and engage in activities beyond local geographical limits. Their online participation expanded to include options from other locations and countries, influencing how they acquire information, feel a sense of belonging, and receive care. Future research should explore the translocal and transnational aspects of older immigrants’ virtual experiences and well-being and how these are influenced by digital infrastructure and services.

The study has several implications for policymaking and service provision to alleviate the impacts of pandemics and public health crises on older immigrants’ spatial mobility. Many participants’ high risk perception and extreme reduction in spatial mobility were not only due to the health consequences of COVID but also related to their language barriers and fear of navigating the healthcare system alone once infected. More culturally inclusive and accessible support for healthcare services, therefore, could alleviate their sense of uncertainty and encourage them to sustain certain levels of outdoor activities. Second, high-density residential neighbourhoods, especially high-rise apartment buildings, require better design of protocols to control the flow of people and prevent high transmission risk situations in public areas. Third, as shown by this study, many older Chinese immigrants initially accessed online resources and activities through their pre-existing social connections. It is critical to draw attention to and develop effective measures to cover older immigrants who are more socially isolated, cultivating their digital literacy and enhancing their capacity to utilize the virtual environment.

This study has several limitations that lead to avenues for future research. First, the online focus group interviews, due to social distancing, limited the sample to participants with a certain level of Internet usage. Internet usage among older Chinese immigrants was observed to greatly increase during the COVID-19 pandemic by this study and previous ones [47,48]. However, conducting interviews online might still attract participants who adapted to virtual activities more quickly, and experienced the virtual environment’s substitute effects more thoroughly. The voices of older Chinese immigrants who lacked the capacity to access digital devices and the Internet were not included in this study. Such older immigrants may nevertheless experience more social isolation and well-being deterioration pertaining to spatial mobility reductions and lack of a substitute channel for everyday well-being-related activities. Future research should apply more diverse data collection methods to explore the experiences of spatial mobility changes and well-being of those with low Internet usage due to lack of digital literacy and access to devices.

In addition, online FG interviews might have entailed fewer interactions among participants compared with in-person ones. Future research could apply in-person FG interviews to gather more insights into how the older Chinese immigrants’ narratives and rationales of spatial mobility changes are formed in relational dynamics. Third, the study focused on Mandarin-speaking older Chinese immigrants, who likely shared similar backgrounds. Future research could include Chinese immigrants from diverse linguistic and cultural backgrounds to explore intra-group differences. Additionally, studies should examine other ethnic groups, such as the growing South Asian community, to identify between-group similarities and differences in spatial mobility patterns during the pandemic. Lastly, the participants’ narratives did not inductively present very strong distinctions of spatial mobility experiences related to the residential locations (urban or suburban), which is often an essential factor identified in empirical studies following the neighbourhood and health framework. Future research can look further into the influences of residential locations on older Chinese immigrants’ mobility during infectious disease pandemics through quantitative deductive measures in Canadian contexts.

## 5. Conclusions

The findings of this study reveal that older Chinese immigrants’ spatial mobility patterns were shaped by their risk perceptions, public health precautions, and the intersecting physical, social and virtual environmental factors during the pandemic. Older Chinese immigrants predominantly had high risk perceptions regarding COVID-19 transmission and a high level of compliance with governmental public health interventions. Their outdoor spatial behaviour was impacted by multiple factors related to the physical and social dimension of their neighbourhood environment, such as types of housing (high rise building vs. detached home), population density, intra-family care, family support, social networks, behavioural cohesion in the neighbourhood, and racial discrimination. The significant reduction in outdoor spatial mobility had direct negative impacts on the overall well-being of many older Chinese immigrants. The virtual environment emerged as a vital channel to sustain the necessary leisure, social, and healthcare-related activities and maintain well-being. It remains unclear, and is worth further investigation, whether the adoption and expansion of virtual environment infrastructure and resources would permanently change the spatial mobility patterns of older Chinese immigrants, and other older adults, in the post-pandemic era. Our study adds to the literature on neighbourhood and health by incorporating the emerging virtual dimension of the broader environment as an important factor shaping older immigrants’ pandemic experience, in addition to the physical/built and social environmental factors. It generates new insight into the changing pattern of spatial mobility, a critical aspect of quality of life and physical and mental well-being for older adults, thus providing timely implications for delivering social support as well as implementing initiatives to enhance the social inclusion and overall well-being of older Chinese immigrants.

## Figures and Tables

**Table 1:** Demographic composition of focus group participants (N = 25).

	Percentage ^1^
Gender	
Female	44%
Male	56%
Age	
65–74	48%
75 and above	36%
Marital status	
Married	72%
Single (including widowed)	24%
Annual income	
<$30,000	76%
$30,000–49,999	4%
50,000 and above	12%
Education	
Secondary and below	12%
Post-secondary	80%
Residential location	
Downtown area	24%
Inner suburban area (e.g., North York)	52%
Outer suburban area (e.g., Thornhill)	16%

^1^ Some categories’ total percentages are not aggregated to 100% due to missing data.

**Table 2:** Major changes in spatial mobility patterns compared with pre-pandemic era.

Spatial Mobility/Outdoor Activity Purposes	Change in Spatial Mobility Compared to Pre-Pandemic	Representative Quotes from Focus Groups
Grocery shopping	Carefully wore personal protection equipment such as masks and gloves and disinfect their purchases; Shortened the shopping time and avoided rush hours to minimize the risks of close contacts with crowds of people; Received delivery from family members and volunteers	“*I go grocery shopping like this: if it opens at 9 am, I will arrive before 9. And I will go and buy what I need, and then leave…. When I arrive home… we have the disinfectant wipes at home (to wipe the purchases)*” (*FG3*, *P4*). “*We are very careful when shopping for groceries. First, we avoid rush hours and go there when there are less people… And we all wear masks and disinfect purchases, right?*” (*FG4*, *P1*). “*We are already 88… So we could call the community [volunteers] to order grocery delivery. In about two months, we will call to request delivery of groceries*” (*FG2*, *P3*).
Leisure activities and physical exercises	Carefully wore personal protection equipment such as masks; Shifted from group activities to individual indoor and outdoor activities and virtual group activities	“*We wash our hands immediately when we come back home. And we wear masks outside*” (*FG1*, *P1*). “*We now practice Tai Chi on our balcony everyday*” (*FG2*, *P3).* “*I basically stay at home and don’t go outside at all… Now I’ve discovered that the Internet is very developed… So I stay in my room day and night. There are many activities online*” (*FG4*, *P3*).
Social and familial activities	Utilized the virtual environment and online communication tools	“*I normally would not visit others. We send messages to one another at most*” (*FG3*, *P4*). “*We used to have meals together, and chat every morning. Once the pandemic came, we stayed at our own homes. And we don’t get together anymore. Sometimes we call each other and talk on WeChat*” (*FG3*, *P1*
Healthcare services	Postponed appointments; Utilized virtual channels: phone calls and online communication tools	“*Now we see doctors online. I only need to call them, and they will tell me (what to do)*” (*FG1*, *P4*). “*My cardiologist told me—because I had had XX procedure—she asked me to come and take a test. But I canceled it with my daughter’s help… The hospital is dangerous (because of COVID-19)*” (*FG4*, *P3*).
Other purposes	Kept distance from others on the street; Avoided taking public transportation	“*When I go for a walk and I see someone coming in my direction, I try my best to avoid them*” (*FG4*, *P2*). “*Buses are closed indoor space and the viruses are easily transmitted in them. So I don’t dare to take the bus now*” (*FG1*, *P2*).

**Table 3:** Influences of the virtual environment on spatial mobility by activity type.

Spatial Mobility/Outdoor Activity Purposes	Change in Spatial Mobility Compared to Pre-Pandemic	Representative Quotes
Grocery shopping	Few older Chinese immigrants shifted to online grocery shopping as many of them found picking produce in-store by hand, by themselves or people they were familiar with (usually their adult children), was necessary to guarantee freshness and quality.	“*For grocery we go and shop by ourselves. Some people suggested that we order delivery. But when the delivery arrives, it is hard to make sure it is fresh. Especially the vegetables… So I don’t think it works*” (*FG4*, *P1*). “*Although it is said that we can buy grocery online… We Chinese are used to shop by ourselves: go to the supermarket and pick produce intentionally. Right?*” (*FG2*, *P2*)
Leisure activities and physical exercises	Many pre-existing leisure and physical exercise group activities were shifted online; meanwhile, new group activities emerged as the pandemic continued.	“*We have an opera class, and we need to sing and rehearse together. And it doesn’t work now. So how can we do it now? We use Zoom, and I find it is okay*” (*Focus group 2*, *Participant #3*). “*And then we have zoom. I would like to participate in Tai Chi activities on zoom*” (*FG3*, *P4*).
Social and familial activities	In-person social and familial activities were mostly cancelled; older Chinese immigrants relied on communication applications to maintain connections.	“*Our life had been very simple, and the major daily schedule was participating in community activities. People got together, chatting and laughing. Now we have changed to online video meetings. It is like the format changed, but the contents did not*” (*FG2*, *P2*).
Healthcare services	After in-person appointments were cancelled, older Chinese immigrants had meetings with their doctors through phone calls and sought virtual care services.	“*Now we see doctors online. I only need to call them, and they will tell me (what to do)*” (*FG1*, *P4*). “*For regular checkups with family doctors, we are doing it through phone calls. But it is very hard to meet with a specialist in-person nowadays*” (*FG4*, *P5*).

## Data Availability

The data presented in this study are available on request from the corresponding author due to the protection of the participants’ anonymity and privacy.
